# Supplementary material for: An Exosomal Urinary miRNA Signature for Early Diagnosis of Renal Fibrosis in Lupus Nephritis
Source: Cells. 2019 Jul 25;8(8):773. doi: 10.3390/cells8080773 (PMC6721515; doi:10.3390/cells8080773)
Supplement: Supplementary file 1 [file cells-08-00773-s001.pdf]

Supporting Information

An exosomal urinary miRNA signature for early diagnosis of renal fibrosis in Lupus Nephritis

Cristina Solé [1], Teresa Moliné[2], Marta Vidal[2], Josep Ordi-Ros[1], Josefina Cortés-Hernández[1]

[1]Hospital Universitari Vall d'Hebron, Vall d'Hebron Research Institute (VHIR), Lupus Unit, Barcelona, Spain; cristina.sole@vhir.org (C.S); jordi@vhebron.net (J.O-R.); fina.cortes@vhir.org (J.C-H.)
[2] Hospital Universitari Vall d'Hebron, Department of Renal Pathology, Barcelona, Spain; teresa.moline@vhir.org (T.M); marta.vidal@vhebron.net (M.V).

INDEX

1. SI Materials and Methods..3

2. SI References……………………………………………………………………………7

2. SI Figures..8

4. SI Tables..21

SI Materials and Methods

Patients and samples

Patients with biopsy-proven active LN were recruited from the Lupus Unit at Vall d'Hebron Hospital (N=45). All patients fulfilled at least 4 of the American College of rheumatology (ACR) revised classification criteria for SLE [1]. Healthy donors were used as controls (N=20). Urine samples were collected from each patient 1 day before renal biopsy and processed immediately to be stored at -80ºC. Patients with urinary tract infection, diabetes mellitus, pregnancy, malignancy and non-lupus-related renal failure were excluded. In addition, key laboratory measurements were obtained including complement levels (C3 and C4), anti-double-stranded DNA (anti-dsDNA), 24-h proteinuria, blood urea nitrogen (BUN), serum creatinine and the estimated glomerular filtration ratio (eGFR) using the Chronic Kidney Disease Epidemiology Collaboration (CKD-EPI) formula [2]. SLE disease activity was assessed by the SLE Disease Activity Index 2000 update (SLEDAI-2Ks; range 0–105) [3]. Patients were classified according to the chronicity index at renal biopsy: low CI (<2, N=18), moderate CI (2-4, N=21), high CI (≥4, N=6). ESRD progression was defined by an estimated glomerular filtration rate (eGFR) < 15 mL/min/1.73 m^2, the initiation of renal replacement therapy or receiving kidney transplantation, or 40% reduction of baseline eGFR [4]. The study was approved by the Vall d'Hebron Ethic Committee and written informed consent was obtained from all patients

Exosome Characterization by Western-Blot, Cryo-TEM and NanoSight analysis:

The protein isolation of exosomes was carried out using the Total Exosome RNA & Protein Isolation Kit (Applied Biosystems). For Western blot analysis, the samples were loaded on 10% polyacrylamide gels and transferred to the membrane. Rabbit anti-TSG101 that is an urinary exosomal marker (dilutions 1:1000, Abcam) was used to incubate the membrane and detected TSG101 exosomal protein using enhanced chemiluminescence detection reagents according to the manufacturer's instructions (Thermo Fischer). TSG101 protein was detected in urinary exosomes (E1-E3) but not in urinary cellular pellet and negative control samples (P1-P3 and C-).

The characterization by Cryo-TEM and NanoSight was done in Universitat Autonoma de Barcelona (UAB). For the Cryo-TEM characterization, a drop of a dilution of exosome pellet (1:100 in PBS) was put on a frozen grid. This was transferred to a cryopreparation chamber (Leica EM CPC) using a GATAN cryotransfer apparatus to prepare the sample to be freeze with propane and ethane. The temperature of the grid was maintained at -174ºC. The vitrified samples were examined using JEM-1400 electron miscroscope with an acceleration voltage of 40 to 120kV. For NanoSight characterization, different dilution of exosome were examined using Nanosight LM-20 particle size analyzer.

Quantification of Urinary exosomes:

The quantification of urinary exosomes was performed using FluorCet Exosome Quantification kit (SBI). After urinary exosome isolation, they were resuspended with 500ul of PBS. We lysis 60ul of urinary exosomes with 60ul Lysis Buffer for 30min at 4ºC. After that, we add for each 96 well plate 50ul of lysed exosome sample or Starndard with 50ul Working stock buffer A and 50ul Working stock buffer B. Incubation was done for 20minutes at room temperature and protected from light. The plate was read using fluorescence plate reader immediately at excitation 530-570nm and emission 590-600nm. For each sample, triplicate determinations were done. To know exactly the quantity of exosome, a standard curve was performed using as standard exosomes quantified by Nanosight analysis (provided for the commercial kit).

RNA extraction from urinary exosomes or pellet:

Lysis process was done by adding to exosomes samples or pellet samples 350µL *Lysis Solution* and 200µL of 95% ethanol. Lysate with ethanol was applied onto the column and centrifuged 14,000g for a minute. Purification was improved by adding 400µL of *Wash Solution* to the column and then centrifuged 14,000g by a minute for 3 times. Finally, the purified RNA bound to the column, was obtained by adding a 50µL of *Elution Buffer* with a brief centrifugation at 200g by 2 minutes to distribute uniformly into the column. Later centrifuge for 1 minute at 14,000g was necessary to obtain the purified RNA samples. These were stored at -80ºC. Degree of RNA quality of different samples was evaluated by using Bioanalyzer PicoChip (exosomes) and NanoChip (pellet).

Small RNA library construction, sequencing and data analysis:

We investigated the miRNA pattern in LN patients by miRNA-seq in the urinary cellular pellet or in the exosome from the same sample (N=3). The library construction was performed using Illumina TruSeq Small RNA Sample Preparation according to the manufacturer's instructions. The 3` and 5` adapters were ligated to the RNA and used as templates for reverse transcription to cDNA. To amplify the obtained cDNA, ligated samples underwent 13 cycles of PCR. For the size selection of amplified cDNA libraries, PCR products were then run on a 6% TBE gel with a custom ladder. The small RNA of approximately 140–160 bp in size was excised from the gel and incubated overnight. The incubated gel was eluted using a spin column. The resulting libraries were subjected to Illumina Hiseq 2000 sequencing platform with 50nt single reads (Illumina, USA). Image analysis, sequencing quality evaluation, and data production summarization were performed using the Illumina/Solexa pipeline. Sequences were analysed for quality control (FASTQC) and aligned to the Human genome (HG19). Aligned sequences were mapped to miRBase_v.21.0 [36]. Reads were normalized to reads per million calculated as follows: Number of sequenced reads/total reads × 1,000,000.

miRNAs expression by qPCR-RT:

Initially a first-strand cDNA synthesis reaction was made to provide template for all microRNA real-time PCR assay using the *miRCURY LNA™ Universal RT microRNA PCR* (Exiqon). Each RNA sample was normalized to a 5ng/µL concentration using nuclease-free water. 2µL was used in combination with a mix of 2µL of *5x Reaction buffer* + 1µL of *Enzyme Mix* + 5µL Nuclease-free water. Reaction was mixed by vortexing gently ensuring the thoroughly mixed of all reagents. Resulting solution was later incubated for 60 minutes at 40º followed by a heat-inactivation of reverse transcriptase for 5 minutes at 95º. Immediately the samples of cDNA were stored at -80º. To avoid process variability the finally samples used for RT-qPCR arrays were obtained by repeating the previous steps threefold and mixing the extracts. All cDNA samples were diluted 1:6.6 in nuclease free water. Every well contains a combination of 5µL of *PCR Master Mix*, 1µL of *PCR primer Mix* and 4µL of diluted cDNA. All analysis were repeated by triplicate. The required amount of primer:master mix solution was calculated and prepared in advance when multiples real-time PCR reactions were performed with same microRNA primer. A 15% of all reagents were included additionally to compensate for pipetting excess material. Real-time PCR amplification was perform following an initial cycle of polymerase activation during 10 minutes at 95ºC. Amplification process consist in 45 amplification cycles of 10 seconds at 95ºC and 1 minute at 60ºC. Finally a dissociation cycle was applied consisting in 15 seconds at 95ªC, followed for 20 seconds at 60º and finally 15 seconds at 95ºC. Transcription was measured by using a ABI PRISM 7000.

Immunofluorescence in renal biopsy:

Immunofluorescence was performed on paraffin-embedded (FFPE) renal biopsies during renal flare (N=3 for each subgroup) using the methodology of Mason *et al* [5]. Slides were baked, soaked in xylene, passed through graded alcohols, and then pre-treated with 10mM

citrate pH 6 in a steam pressure cooker (Decloaking Chamber; BioCare Medical, Walnut Creek, CA) as per manufacturer's instructions. All further steps were done at room temperature in a hydrated chamber. Slides were then treated with peroxidase block (DAKO) for 5 min to quench endogenous peroxidase activity. Staining was performed with 1:50 rabbit anti-SP1 (Abcam, ab124804), 1:100 mouse anti-COL1A1 (Abcam, ab6308) or 1:100 mouse anti-COL4A1 (Abcam, ab6311) overnight at 4ºC. Where indicated, double staining was performed. For immunofluorescent staining, slides were washed in 50mM Tris-HCl (pH 7.4) then labelled with diluted 1:250 Alexa 488 donkey anti-rabbit (Abcam, ab150061) and Alexa 647 goat anti-mouse (Abcam, ab150119) for 2 hours at room temperature and coverslipped using Fluoromount-G with DAPI (Invitrogen, Thermo Fisher Scientific, Carlsbad, CA). Finally using an Olympus BX61 motorized upright microscope with fluorescence and phase optics for immunofluorescence imaging were all the tissue sections visualized.

Evaluation of immunofluorescence and immunohistochemistry

Results were evaluated on blinded specimens by the Vall d'Hebrón pathologist unit under the supervision of the nephropathologist (Dr.Marta Vidal). The percentage of cells expressing the different probes was scored semiquantitatively as follows: 0 (no expression), 1 (11-20%), 2 (40-60%), or 3 (>80%). Staining intensity was scored semiquantitatively as 0 (no staining), 1 (weakly positive), 2 (moderately positive), or 3 (strongly positive).

Overexpression of miR-21/miR-150 and inhibition of mir-29c in human kidney cells

Cells plated on 24-well plates were transfected with mimic miR-21 and mimic miR-150 (Thermo Fisher) or with miR-29c inhibitor (Thermo Fisher Scientific) using Lipofectamine RNAiMAX (Invitrogen) following the manufacturer's instructions. After 48hours, cells were stimulated with TGFβ1 citokine (10 ng/mL, Thermo Fisher Scientific). After 24 hours, the total RNA was extracted using miRCURY RNA Isolation Kit (Exiqon). RNA concentration was obtained by Nanodrop and it was stored at -80ºC for further use. Relative gene expression was measured by qPCR-RT (Applied Biosystem, Table S2). For immunofluorescence, cells were plated on glasses. After transfection and stimulation, they were PBS-washed and fixed with 4% paraformaldehyde during 20min. After washing with PBS, they were permeabilised with 0.1% Triton for 10min. Blocking using PBS 5% BSA was performed during 1hour at room temperature. Primary antibodies were incubated overnight at 4ºC (rabbit anti-SP1 and goat anti-COL1A1, 1:250, Abcam). Secondary antibodies were incubated during 2.5h at room temperature (Alexa-488-conjugated anti-rabbit IgG and Alexa-680-conjugated anti-goat IgG, 1:500, Abcam). DAPI was used to visualise the nucleus cells.

Statistical analysis

Raw threshold cycle (Ct) values were imported from ABI7000 SDS software and relative expression levels for each mRNA were calculated using the comparative Ct method. The data were tested for normality distribution prior to statistical analyses. Non-normally distributed continuous variables were expressed as mean ± SD and compared using non-parametric tests. The clinical/demographic variables of the cases and controls were compared as follows; independent-samples T test for continuous variables and chi-square test for categorical variables. The mean expression of miRNA levels was compared using Mann-Whitney U/Kruskal-Wallis H tests, as appropriate. The relationship between miRNA expression and histological/clinical parameters of LN patients were analyzed using the Spearman correlation coefficient. Risk of progression to ESRD and renal survival rate across urinary exosomal miRNAs were analyzed and compared using the Kaplan-Meier analysis and the log-rank test. The diagnostic performance of biomarkers was evaluated by calculating their sensitivity and specificity using receiver operating characteristic (ROC) curves. Cutoff values were determined according to Youden's index. A combinatorial

analysis of multiple biomarker signatures was carried out using the CombiROC method [6]. This determines optimal combinations of biomarkers through a combined analysis of ROC curves, considering the sensitivity (SE) and specificity (SP) of all possible markers. It is implemented as a freely available web application (http://CombiROC.eu). Statistical analyses were performed by GraphPad Prism version 6 (GraphPad Software, La Jolla, CA, USA) and SAS version 9.3 (SAS Institute Inc., Cary, NC, USA). A p value <0.05 was considered statistically significant.

SI References

1. Hochberg MC. Updating the American college of rheumatology revised criteria for the classification of systemic lupus erythematosus. *Arthritis Rheum*, **1997**, 40, 1725. [doi.: 10.1002/1529-0131(197709)40:9<1725::AID-ART29>3.0.CO;2-Y].

2. Coresh, J.; Turin, TC.; Matsushita, K.; Sang, Y.; Ballew, SH.; Appel, LJ.; Arima, H.; Chadban, SJ.; Cirillo, M.; Djurdjev, O.; et al. Decline in estimated glomerular filtration rate and subsequent risk of end-stage renal disease and mortality. *JAMA*, 2014, 311, 2518–2531. [doi: 10.1001/jama.2014.6634].

3. Gladman, DD.; Ibanez, D.; Urowitz, MB. Systemic lupus erythematosus disease activity index 2000. *J Rheumatol,* **2002**, 29, 288-291. [PMID: 11838846]

4. Levey, AS; Stevens, LA; Schmid, CH; Zhang, YL.; Castro, AF 3rd; Feldman, HI.; Kusek, JW.; Eggers, P.; Van Lente, F.; Greene, T.; et al. A new equation to estimate glomerular filtration rate. *Ann Intern Med,* **2009**, 150, 604-12. [PMID: 19414839].

5. Mason, DY.; Micklem, K.; Jones, M. Double immunofluorescence labelling of routinely processed paraffin sections. *J Pathol*, **2000**, 141, 452-61. [doi: 10.1002/1096-9896(2000)9999:9999<::AID-PATH665>3.0.CO;2-O].

6. Mazzara, S.; Rossi, RL.; Grifantini, R.; Donizetti, S.; Abrignani, S.; Bombaci, M. CombiROC: an interactive web tool for selecting accurate marker combinations of omics data. *Sci Rep*, **2017**, 7, 45477. [doi: 10.1038/srep45477].

Supplementary Figure 1

Figure S1. miTarget 3`UTR miRNA target Clones for Luciferase assay. Vector backbone of miTarget miRNA 3' SP1 UTR and putative binding sites of miR-29c, miR-21 and miR-150 in the SP1 3'-UTR regions.

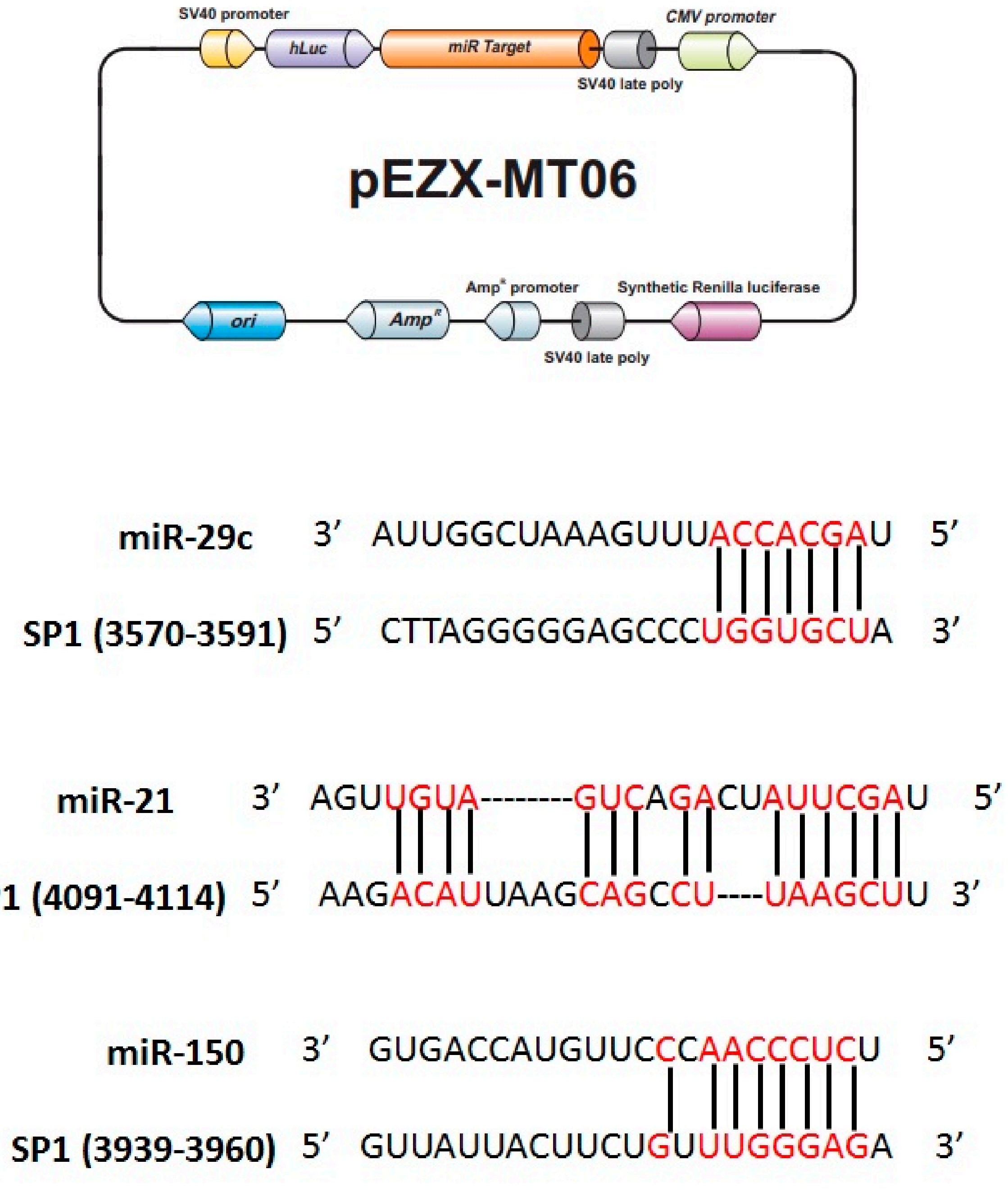

Supplementary Figure 2

Figure S2. Renal chronicity was determined by Gömöri trichrome staining from the paraffin-embedded renal samples. Photomicrographs of the renal lupus nephritis biopsy with Gömöri trichrome staining (20x).

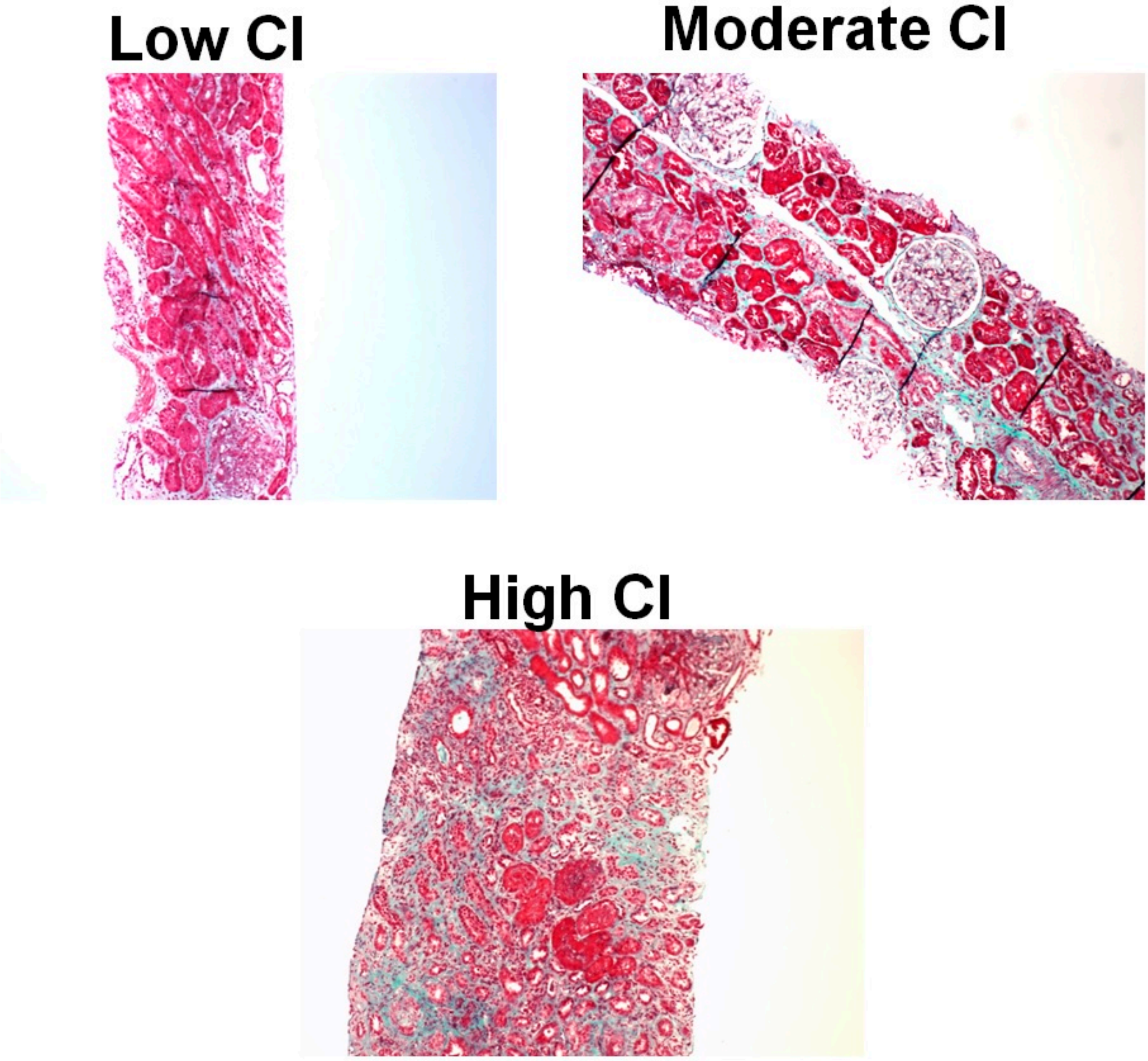

Supplementary Figure 3

Figure S3. Quantification of urinary exosome. Fresh urine (50 mL) was obtained from patients with biopsy-proven active LN to obtain urinary exosomes and they were quantified using FluorCet Exosome Quantitation Kit. No significant differences were observed between CI groups.

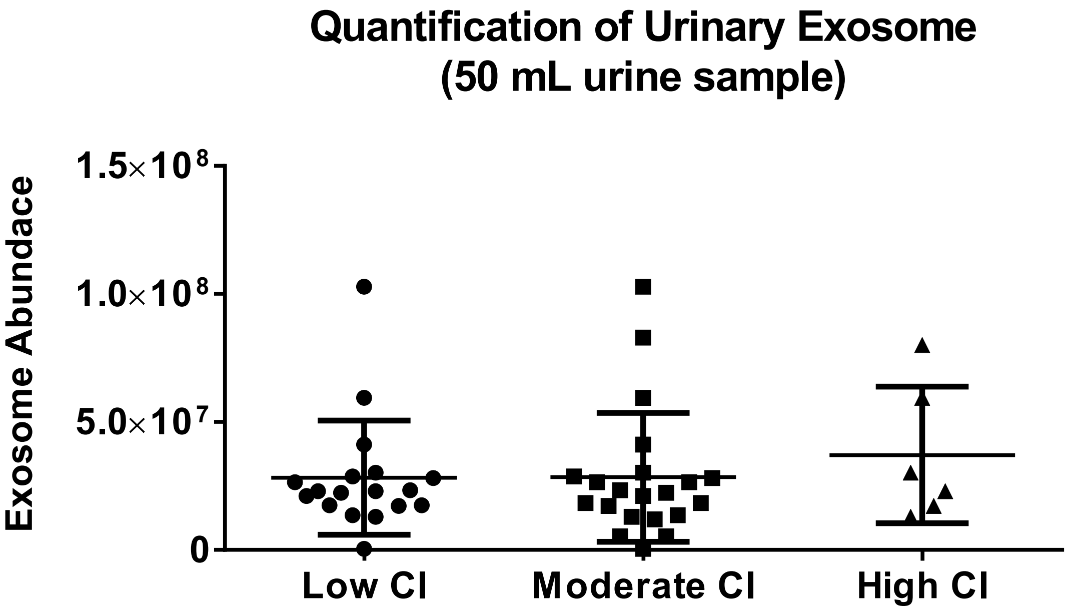

Supplementary Figure 4

Figure S4. Comparation source between urinary exosomal preparations and cellular pellet. rinary exosomal miR-200 levels in lupus nephritis and healthy controls. A) Small RNA libraries wre assessed using the Agilent 2100 Bioanalyzer to obtain the miRNA-seq. Highest percentatge of miRNA was obtained from exosomes in comparison with cell pellet. B) Expression levels of studied miRNAs was evaluated by qPCR-RT in the two source and only in urinary exosomes were detected.

A

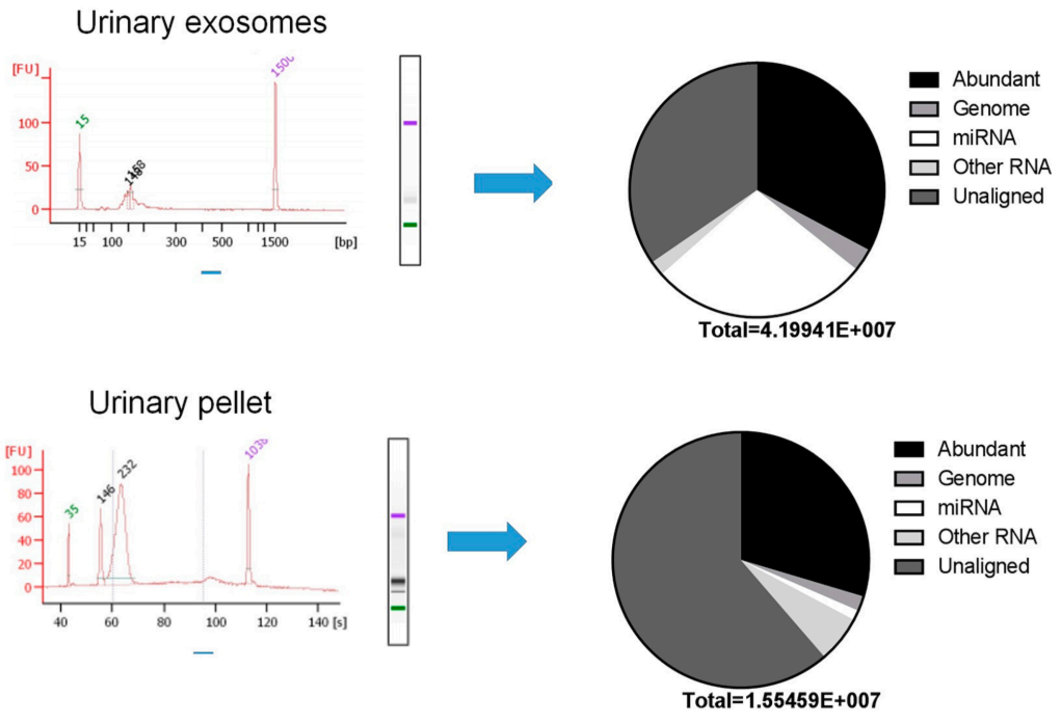

B

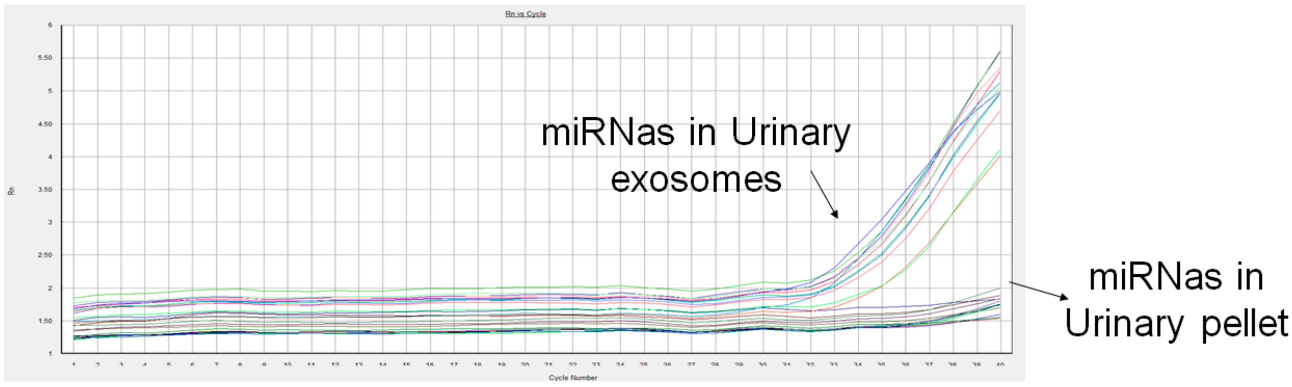

Supplementary Figure 5

Figure S5. Urinary exosomal miR-200 levels in lupus nephritis and healthy controls. No significant differences were observed between the two groups.

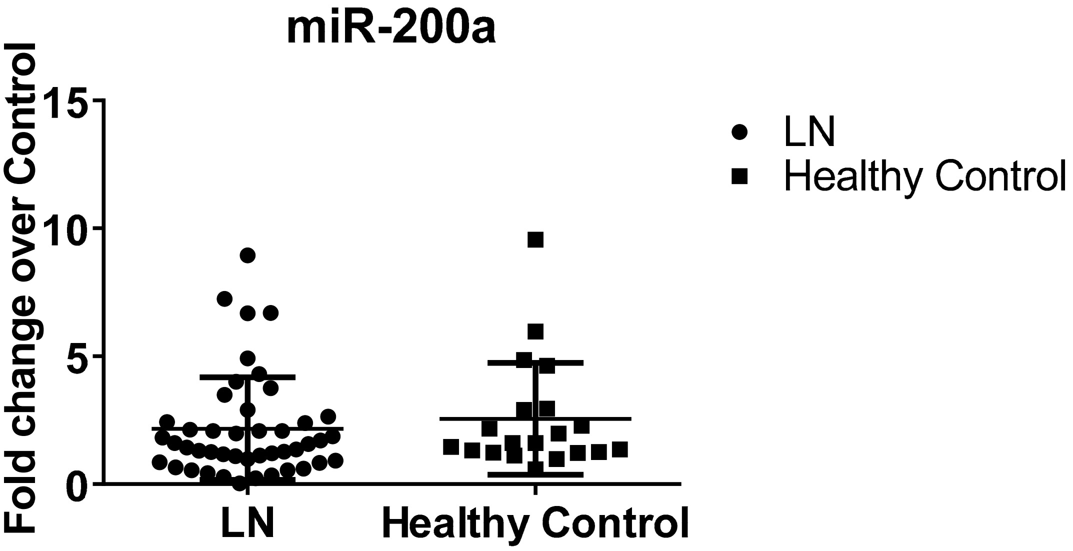

Supplementary Figure 6

Figure S6. Urinary exosomal miR-410 levels in CI subgroup lupus nephritis patients. No significant differences were observed between Low CI, Moderate CI and High CI group.

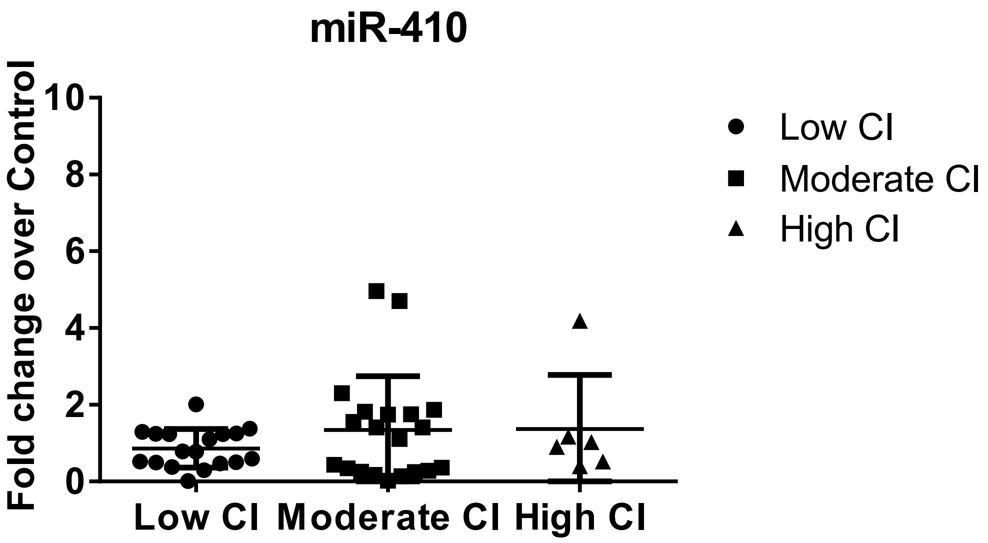

Supplementary Figure 7

Figure S7. Correlation between levels of urinary exosomal miRNAs and chronicity index in lupus nephritis patients. Significant inversely correlation was found with miR-29c expression levels but a positive correlation was found between miR-21 and miR-150.

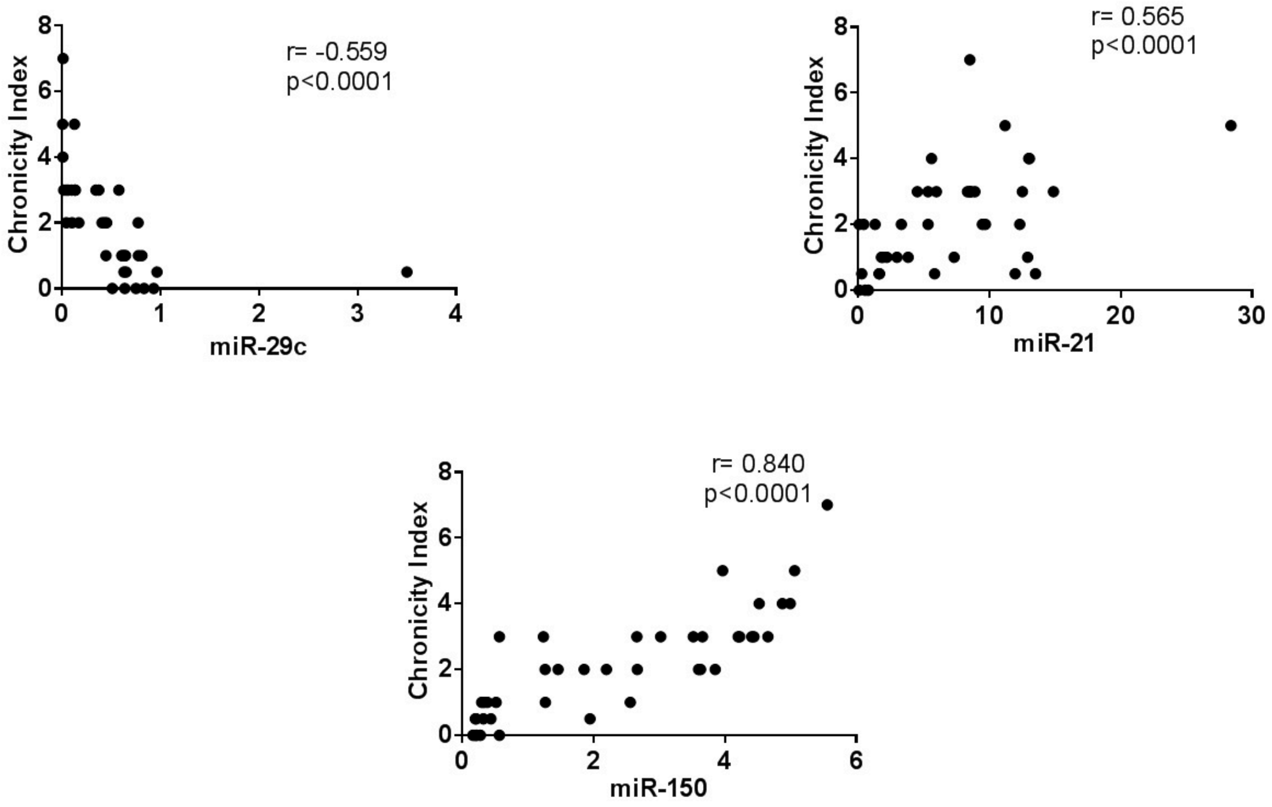

Supplementary Figure 8

Figure S8. Correlation between levels of urinary exosomal miRNAs and tubular atrophy in lupus nephritis patients. Significant inversely correlation was found with miR-29c expression levels but a positive correlation was found between miR-21 and miR-150.

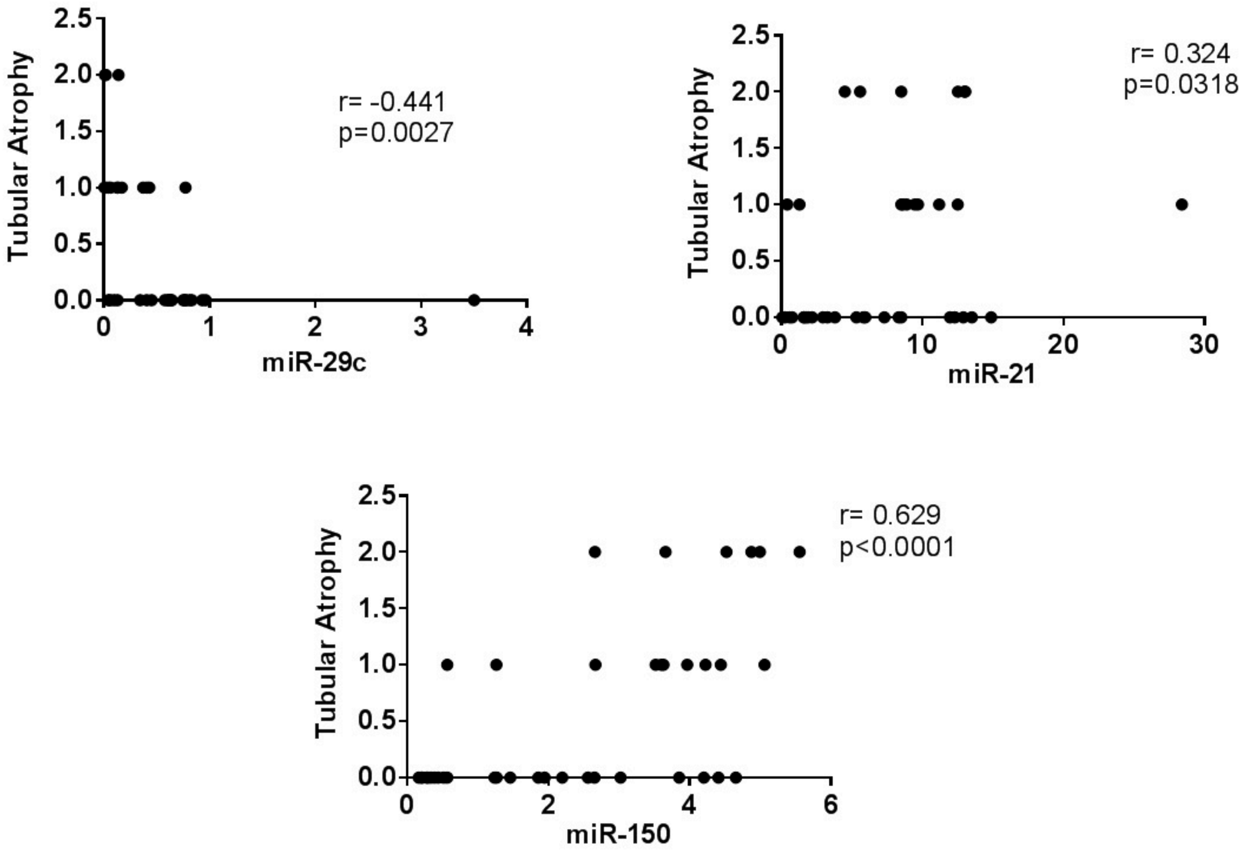

Supplementary Figure 9

Figure S9. Correlation between levels of urinary exosomal miRNAs and intersticial fibrosis in lupus nephritis patients. Significant inversely correlation was found with miR-29c expression levels but a positive correlation was found between miR-21 and miR-150.

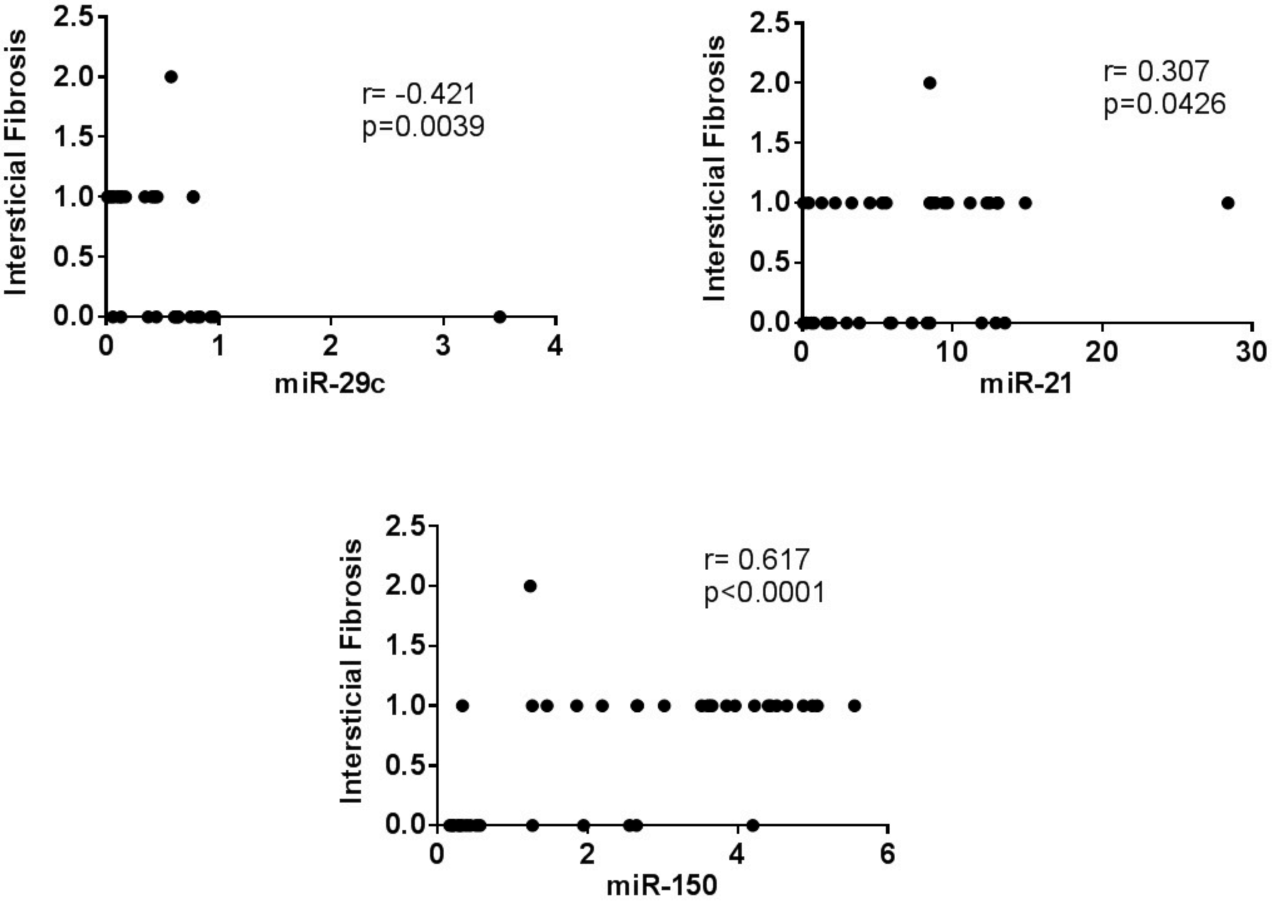

Supplementary Figure 10

Figure S10. Correlation between levels of urinary exosomal miRNAs and glomerular sclerosis in lupus nephritis patients. Significant inversely correlation was found with miR-29c expression levels but a positive correlation was found between miR-21 and miR-150.

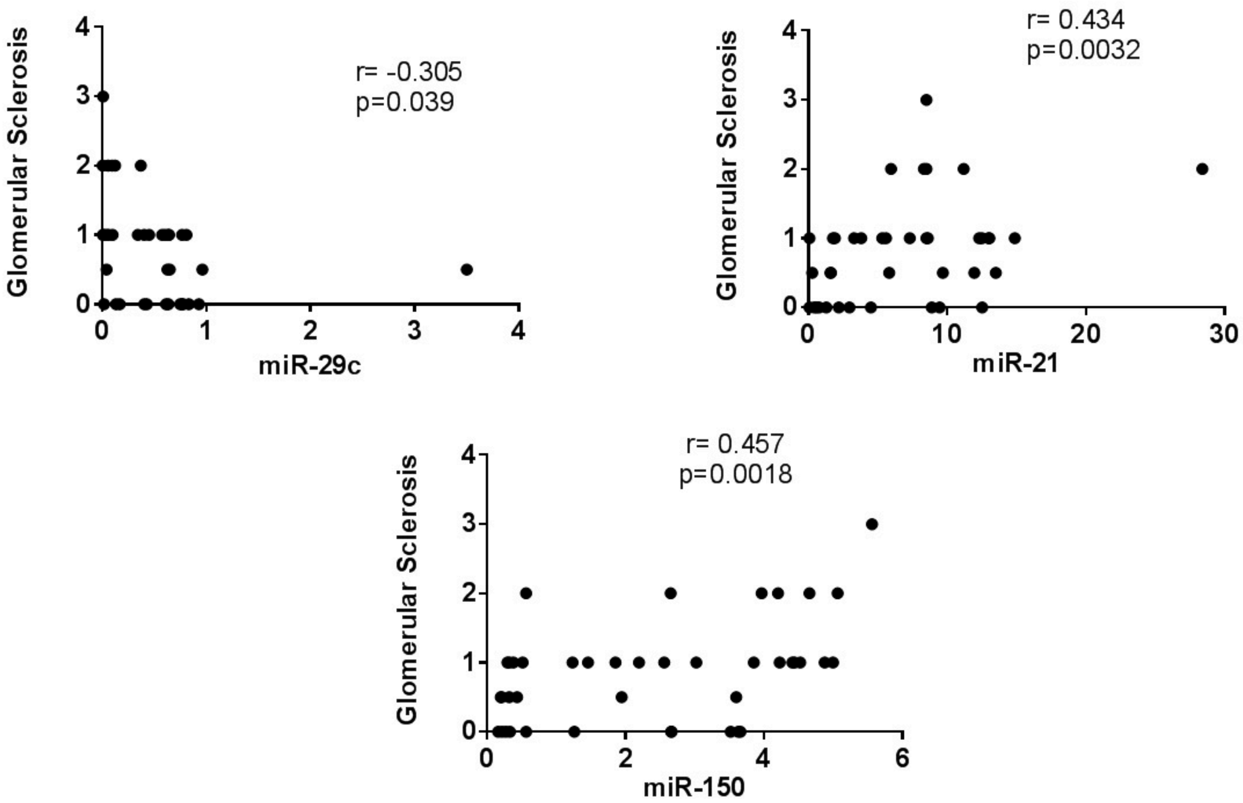

Supplementary Figure 11

Figure S11. Correlation between levels of urinary exosomal miRNAs and fibrous crescents in lupus nephritis patients. Significant inversely correlation was found with miR-29c expression levels but a positive correlation was found between miR-21 and miR-150.

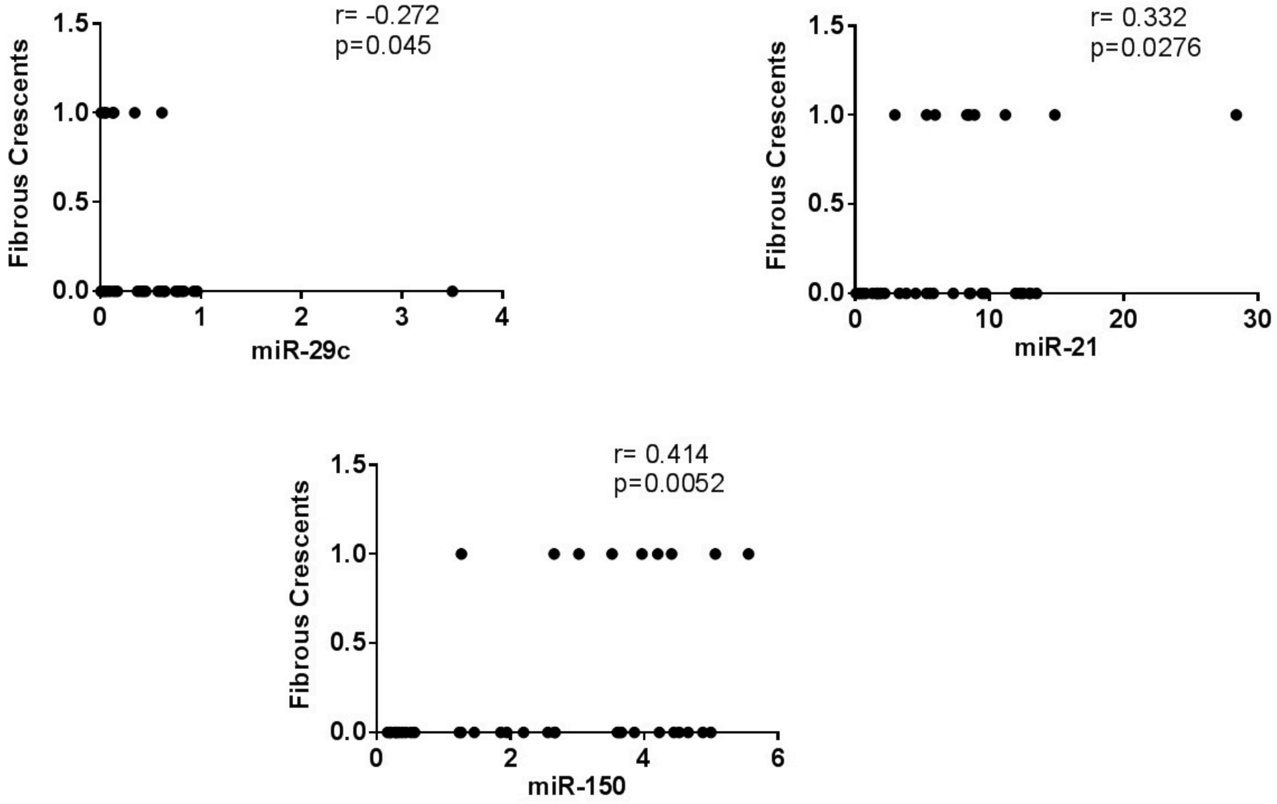

Supplementary Figure 12

Figure S12. Pie chart of miR-29c/miR-21/miR-150 multimarker panel showing the fraction of predictions. FN: false negative. FP: false positive. TN: true negative. TP: true positive.

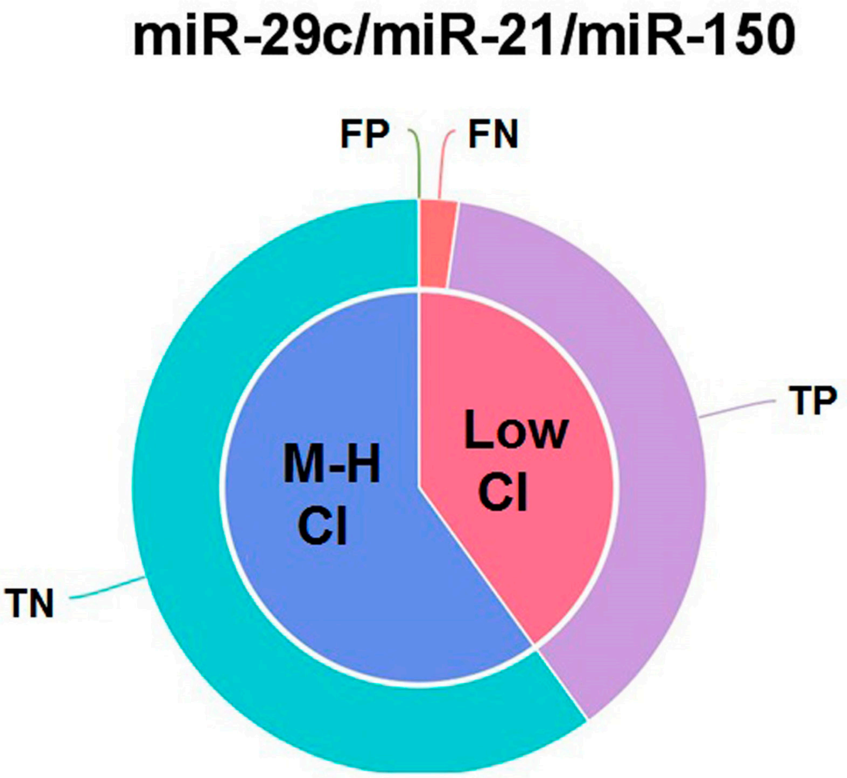

Supplementary Figure 13

Figure S13. Immunohistochemistry of VEGFA in Lupus Nephritis kidney tissues. VEGFA staining was predominantly in the glomeruli and in the tubular structures. Not significant difference were observed between the CI subgroups. It is not a correlation between VEGFA protein and the degree of chronicity index.

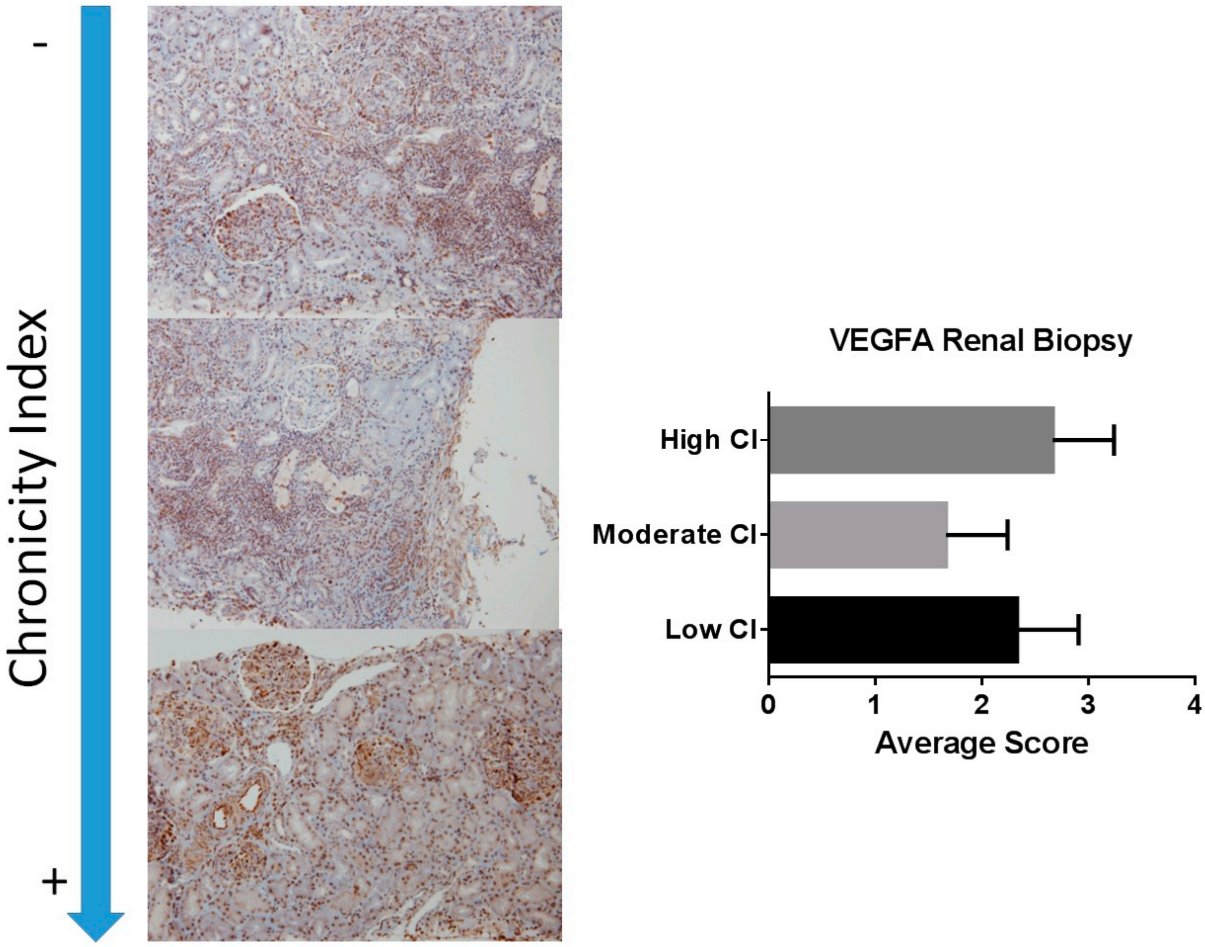

Supplemental Table S1

Table S1. MiRCURY LNA primer IDs from Exiqon

MiRNAs	Primer ID	Sequence 5’-3’
hsa-miR-29c	MIMAT0000681	UAGCACCAUUUGAAAUCGGUUA
hsa-miR-21	MIMAT0000076	UAGCUUAUCAGACUGAUGUUGA
hsa-miR-150	MIMAT0000451	UCUCCCAACCCUUGUACCAGUG
hsa-miR-200a-3p	MIMAT0000707	UAACACUGUCUGGUAACGAUGU
hsa-miR-410	MIMAT0000042	AAUAUAACACAGAUGGCCUGU
RNU6-1	11278	CACGAATTTGCGTGTCATCCTT

Supplemental Table S2

Table S2. Primers IDs used in Taqman RT-PCR from Applied Biosystem

Gene	Primer ID
Smad3	Hs00969210_m1
TGFβ1	Hs00998133_m1
COL1A1	Hs00164004_m1
COL4A1	Hs00266237_m1
SP1	Hs00916521_m1
GAPDH	Hs02786624_g1

Supplemental Table S3

Table S3. Baseline characteristics of LN patients for miRNA-seq analysis.

	Lupus nephritis (n=3)
Demographic	
**Age, yr**	29 ± 3
**Sex, male/female**	0/3
Race/ethnicity, n (%)	
**Caucasian**	3
**Hispanic**	0
Laboratory parameters	
**Serum creatinine, mg/Dl**	1,0 ± 0,4
**eGFR (mL/min)**	90,3 ± 27,1
**BUN (mmol/l)**	26,6 ± 3,7
**Anti-dsDNA Abs, IU/mL**	112 ± 55
**Serum C3, mg/dL**	61,2 ± 12,5
**Serum C4, mg/dL**	14,6 ± 5,5
**Proteinuria, g/24 h**	3,5 ± 2,6
Disease index (SLEDAI-2K)	
**Total SLEDAI score**	11 ± 3
Renal Biopsy, n (%)	
Class, n (%)	
**III**	0
**IV**	3
**V**	0
**Activity Index**	4 ± 3,4
**Chronicity Index**	2,6 ± 0,6

Supplemental Table S4

Table S4. Two biopsies were done for seven patients and their miR-29c, miR-150, miR-21 relative expressions in urinary exosomes were analyzed.

Patient	Renal Biopsy	CI	AI	Class	eGFR	Creatinine	Proteinuria, g/24h
1	Baseline	0,5	2	IV	46	1,87	0,954
	Repeat	3	6,00	IV	89	1,95	1,581
2	Baseline	3	4	IV	83	1,05	5,491
	Repeat	5	7	IV	95	1,12	4,586
3	Baseline	2	7,00	III	89	0,99	1,635
	Repeat	7	2	IV	84	1,04	2,061
4	Baseline	0,5	9,00	III	113	0,72	1,635
	Repeat	1	5	III	112	0,89	2,407
5	Baseline	1	7	IV	63	1,09	7,797
	Repeat	4	6	IV	34	2,18	5,491
6	Baseline	0,5	13	IV	113	0,67	1,591
	Repeat	4	12	IV	122	0,59	2,090
7	Baseline	1	10	IV	100	0,74	3,533
	Repeat	2	0	V	99	0,87	3,680

CI, chronicity index score, consists of the sum of individual scores of four features, including glomerular sclerosis, fibrous crescents, tubular atrophy, and interstitial fibrosis. AI, activity index score, consists of the sum of individual scores of two features, including glomerular activity and tubulointerstitial activity. The maximum score was 12 points for each. eGFR, estimated glomerular filtration rate.

Supplemental Table S5

Table S5. Target genes for miR-29c-3p, miR-21-5p and miR-150-5p validated minimum for two methodology (miRTarBase database).

Target gens of miR-29c-3p
ADAM12; AKT2; AKT3; AMFR; BACE1; BCL2; CCND2; CD274; CD276; CDC42; CDK6; CNOT6; COL15A1; COL1A1; COL1A2; COL3A1; COL4A1; COL4A2; COL6A2; COL6A2; CREB5; CRYBG1; CTNND1; CTSK; DNMT3A; DNMT3B; DSC2; EMP1; FBN1; FGA; FGB; FGG; GAPDH; HMGCR; ITGA6; ITGB1; KLF4; LAMC1; LAMC2; MCL1; MMP15; MMP24; MMP24; MXD1; NASP; PER1; PHACTR2; PPP1R13B; PTEN; RCC2; RFX7; RIOK3; SIRT1; SNX24; SP1; SPARC; SRSF10; TARBP1; TDG; TGIF2; TIAM1; TMEM132A; TUBB2A; VEGFA; WDR26
**65 GENS TARGETS**

Target gens of miR-21-5p
ABCB1; ACBD5; AKT2; ANKRD46; ANP32A; APAF1; APPL1; AUTS2; BASP1; BCL10; BCL2; BMI1; BMPR2; BTG2; CASC2; CASP8; CBX4; CCL20; CDC25A; CDK2AP1; CDK6; CEBPB; COL4A1; COX2; DAXX; DDAH1; DERL1; DNM1L; DOCK4; DOCK5; DOCK7; DUSP10; E2F1; EGFR; EIF4A2; ERBB2; FAM3C; FAS; FASLG; FBXO11; FMOD; FOXO1; FZD6; GAS5; GDF5; GID4; GLCCI1; HIPK3; HMGB1; HNRNPK; HOXA9; HPGD; ICAM1; ICOSLG; IL12A; IL1B; IRAK1; ISCU; JAG1; JMY; KLHL15; KLHL42; LATS1; LRP6; LRRFIP1; MAP2K3; MARCKS; MSH2; MSH6; MTAP; MYC; MYD88; NAV3; NCAPG; NCOA3; NFIB; NTF3; PAG1; PBX1; PCBP1; PCGF2, PDCD4; PELI1; PIAS3; PIK3R1; PLAT; PLOD3; PPARA; PPIF; PSMD9; PTEN; PTPN14; RAB22A; RASA1; RASGRP1; RDH11; RECK; REST; RFFL; RHOB; RMND5A; RP2; RPS7; RTN4; SAR1A; SASH1; SATB1; SECISBP2L; SERPINB5; SERPINI1; SESN1; SETD2 SGK3; SLC16A10; SLMAP; SMAD7; SMARCA4; SMN1; SOCS1; SOCS5; SOCS6; SOX2; SOX5 SP1; SPRY2; ST6GAL1; STAT3; STUB1; TAP1; TGFB1; TGFB2; TGFBR2; TGFBR3; TGFI; TGIF1; TIAM1; TICAM2; TIMP3; TLR3; TM9SF3; TNFRSF10B; TNPO1; TNRC6B; TOPORS; TOR1AIP2; TP53BP2; TP63; TPM1; TPRG1L; TRAF7; UBE2N; VEGFA; VHL; WWP1; YOD1
**155 GENS TARGETS**

Target gens of miR-155-5p
ADIPOR2; AIFM2; ARRB2; BIRC5; CAST; CBL; CCR6; CISH; CNST; COL1A1; COL4A1; CREB1; EGR2; EP300; EREG; FOPNL; MMP14; MUC4; MYB; NANOG; P2RX7; PDIA6; POLD3; PRKCA; SLC2A1; SP1; SRCIN1; SSSCA1; STAT1; STAT5B; SYNPO2; TOM1; TP53; TRPS1; VEGFA; ZEB1; ZNF350
**37 GENS TARGETS**

Supplemental Table S6

Table S6. MicroT-CDS predicted interactions for miR-29c-3p, miR-21-5p and miR-150-5p in *"ECM-receptor interaction"* (KEGG pathway enrichment).

hsa-miR-29c-3p

#	Gene Name	Gene Ensembl id	Interactions	Score	Experimentally Supported
**1.**	**COL4A5**	ENSG00000188153	see interaction	1.000	Yes
**2.**	**COL7A1**	ENSG00000114270	see interaction	1.000	Yes
**3.**	**COL19A1**	ENSG00000082293	see interaction	0.985	Yes
**4.**	**COL3A1**	ENSG00000168542	see interaction	1.000	Yes
**5.**	**COL9A1**	ENSG00000112280	see interaction	0.976	No
**6.**	**ADAMTS2**	ENSG00000087116	see interaction	0.998	Yes
**7.**	**MMP16**	ENSG00000156103	see interaction	1.000	Yes
**8.**	**COL2A1**	ENSG00000139219	see interaction	1.000	No
**9.**	**COL15A1**	ENSG00000204291	see interaction	0.999	Yes
**10.**	**COL4A2**	ENSG00000134871	see interaction	0.866	Yes
**11.**	**COL5A1**	ENSG00000130635	see interaction	0.978	Yes
**12.**	**COL1A1**	ENSG00000108821	see interaction	0.966	Yes
**13.**	**COL4A3**	ENSG00000169031	see interaction	0.970	No
**14.**	**COL4A4**	ENSG00000081052	see interaction	0.999	No
**15.**	**COL1A2**	ENSG00000164692	see interaction	0.989	Yes
**16.**	**COL11A1**	ENSG00000060718	see interaction	0.989	No
**17.**	**COL6A3**	ENSG00000163359	see interaction	1.000	Yes
**18.**	**COL4A6**	ENSG00000197565	see interaction	0.953	No
**19.**	**COL8A1**	ENSG00000144810	see interaction	0.999	No
**20.**	**COL25A1**	ENSG00000188517	see interaction	0.945	No
**21.**	**COL5A3**	ENSG00000080573	see interaction	1.000	Yes
**22.**	**COL5A2**	ENSG00000204262	see interaction	0.986	Yes
**23.**	**COL4A1**	ENSG00000187498	see interaction	1.000	Yes

hsa-miR-21-5p

#	Gene Name	Gene Ensembl id	Interactions	Score	Experimentally Supported
1.	**MMP16**	ENSG00000156103	see interaction	0.807	No
2.	**COL4A1**	ENSG00000187498	see interaction	0.849	Yes

hsa-miR-150-5p

#	Gene Name	Gene Ensembl id	Interactions	Score	Experimentally Supported
1.	**COL1A1**	ENSG00000108821	see interaction	0.895	No
2.	**COL4A4**	ENSG00000081052	see interaction	0.898	No
